# El autocuidado de salud en adolescentes del interior argentino

**DOI:** 10.31053/1853.0605.v80.n4.41091

**Published:** 2023-12-26

**Authors:** Claudia Mariela Nievas, Bernardo J Gandini, Eugenia Toledo, Jhoanna Albornoz

**Affiliations:** 1 Universidad Nacional de Cordoba (UNC); 2 Universidad Nacional de La Rioja (UNLAR)

**Keywords:** autocuidado, adolescente, política de salud, self care, adolescent, health policy, autocuidado, adolescente, política de saúde

## Abstract

**Introducción:**

La salud adolescente transcurre por un grave deterioro en la actualidad. El objetivo de este trabajo fue identificar las prácticas de autocuidado de salud en adolescentes escolarizados entre 15 y 18 años que asisten a escuelas secundarias de cuatro departamentos del interior de la provincia de La Rioja, argentina.

**Metodología:**

estudio descriptivo transversal y analítico; datos relevados entre octubre y noviembre 2022 por muestreo no probabilístico por conveniencia, con análisis de frecuencias absolutas, relativas, análisis multivariado de regresión logística y Odds ratio con IC 95% significancia estadística de p= ˂0,05. Análisis factorial exploratorio (AFE) aplicando como extracción, el método de Componentes principales y como rotación el método Ortogonal Varimax. Software de análisis, SPSS versión 26.

**Resultados:**

Existen discordancias entre la auto-percepción del cuidado de salud y la práctica del cuidado. Se identifican disparidades del autocuidado según el género. Los factores que incrementan el autocuidado son el uso de aplicaciones digitales en salud, los controles médicos anuales o en menor tiempo, el mantener hábitos de pensamientos positivos. La principal preocupación de los adolescentes en relación al autocuidado de salud fue el consumo de bebidas con contenido etílico.

## Abstract (en)

**Introduction:**

Adolescent health is currently undergoing a serious deterioration. The aim of this study was to identify the self-care practices in adolescents between 15 and 18 years of age attending secondary schools in four departments of the interior of the province of La Rioja, Argentina.

**Methodology:**

descriptive cross-sectional and analytical study; data collected between October and November 2022 by non-probabilistic convenience sampling, with analysis of absolute and relative frequencies, multivariate logistic regression analysis and Odds ratio with 95% CI statistical significance of p= ˂0.05. Exploratory factor analysis (EFA) using the Principal Components method as extraction and the Orthogonal Varimax method as rotation. Analysis software, SPSS version 26.

**Results:**

There are discordances between the self-perception of health care and the practice carried out by the adolescents. Gender disparity in self-care is identified. The factors that increase self-care are the use of digital health applications, annual or shorter medical check-ups, and maintaining positive thinking habits. The main concern in relation to self-care among adolescents was the consumption of alcoholic beverages.

## Resumo

**Introdução:**

A saúde dos adolescentes está actualmente a sofrer uma grave deterioração. O objectivo deste estudo foi identificar práticas de autocuidado em adolescentes entre os 15 e 18 anos de idade que frequentam escolas secundárias em quatro departamentos do interior da província de La Rioja, Argentina.

**Metodologia:**

estudo descritivo e analítico transversal; dados recolhidos entre Outubro e Novembro de 2022 por amostragem de conveniência não-probabilística, com análise de frequências absolutas e relativas, análise multivariada de regressão logística e odds ratio com 95% de significância estatística CI de p= ˂0.05. Análise de factores exploratórios (EFA) utilizando o método dos Componentes Principais como extracção e o método Orthogonal Varimax como rotação. Software de análise, SPSS versão 26.

**Resultados:**

Existem discrepâncias entre a auto-percepção dos cuidados e a prática dos cuidados. São identificadas disparidades de género no autocuidado. Os factores que aumentam os autocuidados são a utilização de aplicações digitais de saúde, check-ups médicos anuais ou mais curtos, e a manutenção de hábitos de pensamento positivo. A principal preocupação dos adolescentes em relação ao autocuidado foi o consumo de bebidas alcoólicas.

**CONCEPTOS CLAVE**

Que se sabe sobre el tema: Un adecuado autocuidado de salud desde edades tempranas, alcanza mayores beneficios de calidad de vida en la etapa adulta. Los jóvenes sostienen una mayor elasticidad al momento de incorporar nuevos patrones de comportamiento más saludables.

Qué aporta este trabajo:Precisa realidades sobre las prácticas de autocuidado de salud de adolescentes entre 15 y 18 años, posibilitando ajustar programas educativos y políticas públicas de promoción de salud, a favor de una mayor calidad de vida poblacional futura.

**Divulgación**

Proteger la salud adolescente anticipa calidad de vida para las próximas generaciones. Existen advertencias a nivel mundial, por el desmejoramiento actual de indicadores de salud adolescente, agudizados a su vez, por la pandemia COVID-19. En este artículo, se estudia la autopercepción de salud que poseen adolescentes entre 15 y 18 años, se identifican factores influyentes en su adhesividad, y como ello se traslada a la práctica frecuente del autocuidado de salud, en adolescentes escolarizados del interior de La Rioja, Argentina

## Introducción

Recientemente Naciones Unidas alertó que existe a nivel mundial un grave deterioro de salud en los adolescentes, con una severa regresión de los indicadores claves del bienestar infantil para alcanzar los Objetivos del Desarrollo Sostenible (ODS) ^1^ . Vacunación insuficiente, pérdida de aprendizaje por causas de la pandemia COVID-19, crisis socio-económicas, y las emergencias climáticas, disminuyen en los adolescentes posibilidades de vida ^2^ . Actualmente en la Región de las Américas, un 16% de su población total tiene entre 10 y 19 años, representando la mayor cohorte en la historia del mundo y Región ^3^ . En Argentina, la proporción de niñas, niños y adolescentes respecto a la población total tiende a disminuir ^4^ . UNICEF por su parte, reportó tendencias crecientes en los adolescentes argentinos del consumo de sustancias a partir del año 2010, como así también, una reducción de los controles de salud y alteraciones de hábitos en inseguridad alimentaria, ansiedad y depresión ^5^ . Estudios indican que uno de cada cuatro estudiantes refirió que en la escuela se provee bebidas azucaradas ^6^ ; que seis de cada diez muertes en adolescentes argentinos ocurren por causas externas (accidentes, homicidios, suicidios y otros eventos violentos) ^7^ , todas causas prevenibles. Respecto a la posición de las políticas públicas argentinas con agenda adolescente, autores identificaron que en ellas existe una fragmentada compartimentalización, inclusive, pese al incremento sostenido del sobrepeso y obesidad, entre otros ^8^ .

En este contexto, resulta trascendental observar las prácticas de autocuidado de salud de adolescentes, definidas como el conjunto de conductas, actividades y sentimientos que realizan y perciben las personas de forma individual o grupal, para favorecer su salud, prevenir o acompañar la enfermedad ^9, 10^ . Cuanto mayor es la práctica del autocuidado, aumenta el sentimiento de satisfacción ^11^ , el nivel de adhesividad a las buenas prácticas, y la toma de decisión para cuidar su propia salud ^12^ . El autocuidado está relacionado con la edad, la escolaridad, la raza, el estado civil, el nivel de ingresos; también incluye a aspectos como el tabaquismo, la actividad física, los hábitos alimentarios, el consumo de alcohol, la presencia de comorbilidad, el peso corporal, entre otros ^13^ . Además, se conoce que la baja alfabetización en salud es considerada una epidemia sanitaria silenciosa, y que ello ocurre asociado al uso deficiente de servicios médicos, mayor tasa de hospitalización ^14^ , conductas de riesgo, uso de drogas en alumnos de escuelas secundarias, mayor uso de dispositivos electrónicos como celular y otras pantallas ^15, 16^ . Asimismo, el entorno sociocultural del adolescente aporta a la construcción de su salud mental y a la toma de decisión de forma positiva o negativa ^17^ , reproduciendo trasformaciones y comportamientos que pueden afectar su salud ^18^ .

## Objetivos

Identificar hábitos de prácticas en autocuidado de salud de adolescentes entre 15 y 18 años que asisten a escuelas secundarias de cuatro departamentos del interior de la provincia de La Rioja, Argentina.

## Materiales y Métodos

Estudio descriptivo, transversal y analítico, a partir de datos primarios. La muestra estuvo conformada por estudiantes adolescentes de ambos géneros, que asisten a seis escuelas secundarias públicas de los departamentos Chamical, Belgrano, Ocampo, y Ángel Vicente Peñaloza, durante setiembre y octubre del año 2022. El muestreo fue no probabilístico por conveniencia. Criterios de inclusión: adolescentes de escuelas secundarias entre 15 y 18 años que otorgaron consentimiento informado para participar de la investigación. Criterios de exclusión: Presentar alguna discapacidad intelectual, barrera idiomática, o situación que imposibilite completar de forma auto administrada la encuesta. Como instrumento se utilizó un cuestionario de 40 ítems, auto-administrado, que indagó características demográficas como edad, género, situación personal de salud, datos sobre prácticas de autocuidado y toma de decisión. Las preguntas fueron construidas para este estudio, adaptadas al contexto socio-cultural, fundamentadas en la Escala bidimensional de Autoestima de Rosenberg (RSES) ^19^ [mide autoestima-autoconfianza-satisfacción personal], y en el cuestionario bidimensional general de salud (GHQ-12) ^20^ [mide funcionamiento-afrontamiento social]. Las encuestas fueron relevadas por personal previamente capacitado, con el acompañamiento de un facilitador asignado por el director de cada establecimiento.

**Variables dependientes:** a) "Prácticas de autocuidado de la salud" definida como poseer conductas concretas en la vida hacia sí mismo y hacia el entorno en beneficio de su vida, salud o bienestar ^21^ , categorizada como poco o muy importante. b) "Género", definida según propia percepción en sentirse varón o mujer ^22^ , categorizada como masculino o femenino.

**Variables independientes:** (definidas por auto-reporte)

*Actividad física:* Efectuar prácticas de actividad física dos veces por semana (si/no).

*Hábito tabáquico:* Poseer hábito de fumar (si/no)

*Uso de aplicación móvil en salud:* definida como utilizar habitualmente en teléfono personal aplicaciones relacionadas con la salud (si/no).

*Ultimo examen médico:* definido como tener un control médico en (± 1 año).

*Alimentación saludable:* definida como tipo y consumo habitual de alimentación (saludable/no saludable).

*Capacidad de modificar hábitos:* definida como poseer elasticidad personal para modificar hábitos y conductas por otras más saludables (si/no).

*Pensamientos positivos:* definida como tener actitud frecuente de pensamientos positivos (si/no).

*Puedo resolver un conflicto fácilmente:* definida como tener aptitud de resolución de conflictos con sus compañeros (si/no).

*Puedo decir que no:* definida como tener capacidad para decir que NO sin que se molesten los demás (si/no).

*Digo lo que pienso al grupo:* definida si expone sus pensamientos con facilidad al grupo (si/no).

*Pienso antes de actuar:* definida como poseer hábitos de pensar antes de actuar (si/no).

*Descanso lo suficiente:* definida por tener el descanso que considera suficiente (si/no).

*Guardo rencor:* definida por tener capacidad de deshacerse fácilmente de pensamientos de rencor (si/no).

### Análisis de datos:

Se utilizaron frecuencias absolutas y relativas, regresión logística multivariada, con intervalo de confianza del 95% y valor de significancia estadística de p˂0,05. Para agudizar búsqueda de dimensiones latentes capaces de explicar el máximo de información contenida en los datos, se aplicó Análisis Factorial Exploratorio (AFE), con determinación de pesos factoriales, comunalidades y de variables jerarquizadas independientes latentes no observables respecto a la dependiente. Como medida de adecuación se consideró al valor determinante de la matriz de correlación, el test de esfericidad de Barlett y la prueba de adecuación de Kaiser-Mayer Olkin (KMO). Para la extracción se utilizó el método de Componentes principales, basado en autovalor >1, y para la rotación de utilizó el método ortogonal Varimax. Se utilizó SPSS versión 26.

### Consideraciones éticas:

El protocolo fue aprobado por revisión de pares de la universidad, y por integrantes del Comité de Capacitación y Docencia de la Zona Sanitaria 5. Asimismo, se respetaron los principios de la declaración de Helsinki, y requerimientos éticos de normativas en investigaciones a nivel internacional, nacional y provincial.

### Limitaciones del estudio:

Este estudio se basa en datos auto informados por los propios adolescentes, implicando ello una limitación del estudio. La fuente de datos primaria auto percibida condiciona profundizar dimensiones y consecuentemente resultados.

## Resultados

De 328 adolescentes que participaron presentaron una media en edad de 16,2 [15-18] (DS 1,23) años, el 59,5% (n=195) correspondieron al sexo femenino y 40,5% (n=133) al masculino. Estaban sanos según autorreporte el 57,6% (n=189) y entre ellos el 69,2% (n=92) eran varones y 49,7% (n=97) mujeres. De los que expresaron tener algún tipo de enfermedad por más de seis meses, la de mayor frecuencia fue migraña en mujeres 19% (n=37) y 9% (n=12) en varones, ansiedad en mujeres 5,6% (n=11) y asma 7,5% (n=10) en varones. Las prácticas de autocuidado de salud indicaron poseerlas con regularidad el 73,5% (n=241), y consideró que puede mejorarla con ayuda de un enfermero el 24,1%, un amigo 19,2%, el médico 17,1%, con información digital 16,8%, de la propia familia 14,3%, por radio y televisión 11,6%. El 13,1% (n=43) tomaba con frecuencia algún tipo de medicamento, el 10,7% (n=35) fuma, y el 87,2% (n=286) utiliza en su móvil algún tipo de aplicación relacionada con la salud. La principal bebida de consumo fue agua 94,5% (n=310), bebidas azucaradas con gas 83,8% (n=275), sin gas 59,2% (n=195). Como problema relevante percibido por los jóvenes se indicó el consumo de alcohol 73,5% (n=241), aunque auto-reportaron consumir cerveza el 13,1%, fernet 18%. El 82,3% (n=270) respondió realizar algún tipo de actividad física dos veces por semana durante al menos 20 minutos, de ellos 87,2% fueron mujeres y 95,1% varones.

**Tabla 1:** Característica general descriptiva de la muestra de estudio (n=328)

Variable	N	Mínimo	Máximo	Media	Desv. Est.		Varianza	
Edad	328	15	18	16,22	1,232		1,517	
Genero según auto-reporte *	328	0	1	0,59	0,492		0,242	
Enfermedad **	328	0	1	0,11	0,336		0,113	
Hábito tabáquico (fuma?) ***	328	0	1	0,11	0,309		0,096	
Utiliza aplicación móvil en salud †	328	0	1	0,13	0,335		0,112	
Ultimo examen médico antes de 1 año ††	328	0	1	0,45	0,498		0,248	
Consumo de alimentos saludables †††	328	0	1	0,52	0,501		0,251	
Practico actividad física semanal ‡	328	0	1	0,62	0,486		0,237	
Puedo adquirir hábitos más saludables ‡‡	328	0	1	0,77	0,421		0,177	
Tengo pensamientos positivos ‡‡‡	328	0	1	0,61	0,488		0,238	
Tengo mis espacios de tiempo ¶	328	0	1	0,81	0,395		0,156	

*0: masculino/1: femenino.

**0: sin enfermedad/1: con enfermedad (signos o síntomas por tiempo mayor de 6 meses).

***0: no fuma/ 1: si fuma tabaco.

†0: no utiliza/1: si utiliza aplicaciones móviles relacionadas a la salud.

††0 último examen médico mayor a 1 año/1: último examen médico realizado hace 1 año o menos.

†††0: no mantengo hábitos alimentarios saludables/1: si mantengo hábitos alimentarios saludables.

‡0: no practico actividad física dos veces por semana/1: si practico actividad física dos veces por semana.

‡‡0: habitualmente no tengo pensamientos positivos/1: habitualmente si tengo pensamientos positivos.

‡‡‡0: no puedo adquirir/modificar mis hábitos por otros más saludables/1: si puedo adquirir/modificar mis hábitos por otros más saludables.

¶0: habitualmente no tengo el espacio de tiempo que necesito/1: habitualmente si tengo el espacio de tiempo que necesito.

### Prácticas de autocuidado de salud

Para identificar factores que explican las "Prácticas de autocuidado de salud", se utilizó Regresión Logística binaria. En modelo ajustado, el resultado explica entre el 17,2 % [R² de Cox y Snell] y 25% [R² de Nagelkerke] de la variable dependiente, y clasifica correctamente en el 76,2% de los casos. El uso de aplicaciones de salud en móviles, el tener capacidad en modificar hábitos por otros más saludables, y el tener pensamientos positivos con frecuencia, incrementan 2,9 veces más la probabilidad de poseer prácticas frecuentes de autocuidado de salud. El realizar controles de salud en tiempo menor de un año, incrementa 2,035 veces más la probabilidad de aplicar prácticas frecuentes de autocuidado de salud. El consumo habitual de alimentos saludables, incrementa 2,42 veces más la probabilidad de efectuar prácticas de autocuidado.

**Tabla 2:** La práctica de autocuidado de salud desde la percepción adolescente

Variables			I.C. 95,0% para OR	
	Sig.	OR	Inferior	Superior
Actividad física (dos veces por semana)	0,998			
Hábito tabáquico (no fuma)	0,733			
Uso de APP en móvil (usuarios frecuentes)	0.006	2,947	1,353	6,416
Ultimo examen médico (menos de 1 año)	0,017	2,035	1,136	3,623
Alimentación saludable (si)	0,003	2,421	1,349	4,347
Capacidad de modificar hábitos (si)	0,001	2,906	1,567	5,376
Pensamientos positivos (si)	0,001	2,932	1,669	5,154
En esta tabla se informa la probabilidad de tener “Practicas de Autocuidado de la Salud” según las siguientes variables				
Variable dependiente: definida por auto reporte la práctica del propio autocuidado de saud, categorizada como poco importante o muy importante.				
Variables independientes:				

Actividad física: definida por auto reporte de tener el hábito semanal en realizar actividad física dos veces por semana, como si o no.

Hábito tabáquico: definida por auto reporte si fuma o no fuma.

Uso de APP móvil: definida por auto reporte su utiliza en su móvil aplicaciones con objetivos de salud como, si utiliza o no utiliza.

Ultimo examen médico: definida por autorreporte como el haber efectuado un control de salud ˂ 1 año o > 1 año.

Alimentación saludable: definida por autorreporte como mantener consumos alimentarios habituales como saludables o no saludables.

Capacidad de modificar conductas por hábitos más saludables: definida como la capacidad auto reportada por el adolescente en tener capacidad de modificar conductas por hábitos más saludables como si o no.

Pensamientos positivos: definida por autorreporte como tener una actitud habitual de pensamientos positivos, como si o no.

**OR. (Razón de Odds). Obtenido mediante un modelo de regresión logística multivariado, p: nivel de significancia estadística ˂0,05 Adolescentes que utilizan APP relacionado con salud en su móvil, tienen 2,9 veces más de probabilidad de tener prácticas de autocuidado de su salud; los que realizaron un control de salud en tiempo ˂ 1 año, tienen 2,035 veces más de probabilidad de tener prácticas de autocuidado de la salud; los que auto reportaron tener hábitos de alimentación saludables, tienen 2,42 veces más de probabilidad, los que indicaron tener capacidad de modificar sus prácticas para adquirir hábitos más saludables tienen 2,9 veces más de probabilidad, y los que auto reportaron tener
**

### La Toma de decisión según el género.

En modelo ajustado se explica entre el 14,3 % [R² de Cox y Snell] y 19,3% [R² de Nagelkerke] de la variable dependiente, y clasifica correctamente el 67,4% de los casos. Las adolescentes exhibieron mayor fortaleza para la toma de decisión. El ser mujer incrementa 2,1 veces más la capacidad de resolución de conflictos en el grupo escolar; incrementa 1,9 veces más la probabilidad de pensar antes de actuar y de poseer suficientes horas de descanso; a su vez tienen 2,4 veces más la probabilidad de deshacerse fácilmente de pensamientos de rencor, que los varones.

**Tabla 3:** Parámetros en la toma de decisión de los adolescentes

Variables	Sig.	Odds Ratio	I.C. 95,0% para OR	
			Inferior	Superior
Capacidad de resolución de conflicto con compañeros(si)	0,023	2,115	1,111	4,027
Puedo decir que NO sin que se molesten los demás (si)	0,119			
Digo lo que pienso al grupo (si)	0.182			
Pienso antes de actuar (si)	0,012	1,979	1,162	3,373
Descanso el tiempo suficiente (si)	0,009	1,902	1,162	3,081
Me resulta fácil deshacerme de sentimientos de rencor (si)	0,001	2.469	1,522	4,001
En esta tabla se informa el nivel de probabilidad del "género femenino" con la "Toma de decisión" en situaciones frecuentes del entorno natural de las adolescentes detalladas en las siguientes variables:				
Variable dependiente: Género, definida por auto reporte en relación al género auto percibido como mujer o varón.				
Variables independientes:				
Puedo resolver un conflicto fácilmente: definida por autorreporte como tener la capacidad para resolver un conflicto con los compañeros valorado como si o no.				
Puedo decir que NO: definida por auto reporte de poseer capacidad para decir que NO sin que se molesten los demás, valorado como si o no.				
Digo lo que pienso al grupo: definida por auto reporte si expone sus pensamientos al grupo con facilidad, valorado como si o no.				
Pienso antes de actuar: definida por auto reporte como el poseer el hábito de pensar antes de actuar, valorado como si o no.				
Descanso lo suficiente: definida por auto reporte tener el descanso que considera suficiente, valorado como si o no.				
Guardo rencor: definida por auto reporte de poseer la capacidad de deshacerse fácilmente de pensamientos de rencor, valorada como si o no.				
OR. (Razón de Odds). Obtenido mediante un modelo de regresión logística multivariado, p: nivel de significancia estadística ˂0,05				
Adolescentes mujeres poseen significativamente mayores fortalezas en la toma de decisión. el ser mujer incrementa 2,1 veces más la capacidad de resolución de conflictos que pueden presentarse en el grupo escolar, incrementan en 1,9 veces más la probabilidad de pensar antes de actuar, tienen 1,9 veces más la probabilidad de poseer horas de descanso consideradas suficientes, y tienen 2,4 veces más la probabilidad de deshacerse de pensamientos de rencor, que los varones.				

### Identificación y ponderación de Factores latentes explicativos

Mediante análisis factorial exploratorio [AFE] se identificaron probables factores latentes. La prueba de adecuación se validó mediante el valor determinante de matriz de datos (0,065), confirmado por el Test de Esfericidad de Bartlett (sig.˂ 0,001) y la medida de Káiser-Meyer-Olkin [KMO] 0,673, valorado como puntuación de coeficiente aceptable. Se aplicó la regla de Káiser (autovalor >1), en extracción el método de Componentes principales, y en rotación el método ortogonal Varimax. De 40 ítems considerados, se extrajeron 5 factores que se encontraban latentes, alcanzando el porcentaje explicativo de 61,33% del total de las varianzas (Tabla 4). La validez interna del constructo, se efectuó mediante sub-escalas medida por alfa de Cron Bach (0,722-0,761).

**Factor 1:** se identificó asociado a tres variables cuyo denominador comun es el consumo de bebidas con contenido alcohólico [vino-fernet-cerveza], asumiendo un poder explicativo del 19,4% de la varianza total. Analizado por cada una de las variables, la referida al consumo de fernet, quedó explicada por el total de los factores en un 74,8% (Comunalidad 0,748), y representa el 70,89% (0,842²=70,89%) de la varianza total, es decir, el 94,77% (0,7089/0,748=94,77%) del total del espacio de los factores. La variable referida al consumo de vino, quedó explicada por el 68,1% (Comunalidad 0,681), del total de los factores, representando el 65,7% (0,811²=65,77%) de la varianza total, es decir, el 96,57% (0,6577/0,681=96,57%) del total del espacio de los factores. En cuanto a la variable referida al consumo de cerveza, quedó explicada por el total de los factores en un 59,6% (Comunalidad 0,596), representando el 58,67% (0,766²=58,67%) de la varianza total, o sea, el 98,43% (0,5867/0,596=98,43) del total del espacio de los factores. En menor medida y en extremo opuesto, se identificó asociación en sentido negativo con la variable sentirse parte del grupo de compañeros (Componente rotado =-0,108), y con la variable considerar que el consumo de alcohol es un hábito negativo (Componente rotado =-0,115). Del análisis de este factor, se determinó que existe asociación entre el adolescente con el consumo de bebidas de contenido alcohólico [variable latente], y que explican a su vez en esta dimensión, una relación inversa con el sentimiento de pertenencia al grupo.

**Factor 2:** Asociada principalmente a dos variables, el considerar principalmente que la familia y en segundo lugar los amigos, pueden apoyarlos en el autocuidado de su propia salud, con un poder explicativo del 13,56% de la varianza total. La variable que se refiere al apoyo familiar, queda explicada por el total de los factores en un 61,3% (Comunalidad 0,613), lo cual representa el 58,52% (0,765²=58,52%) de la varianza total, es decir, el 95,46% (0,5852/0,613=95,46%) del total del espacio de los factores. La variable que se refiere al apoyo de los amigos, queda explicada por el total de los factores en un 60,5% (Comunalidad 0,605), y representa el 59,59% (0,772²=59,59%) de la varianza total, es decir el 98,49% (0,5959/0,605=98,49%) del total del espacio de los factores. Del análisis de esta segunda dimensión se identificó que el principal sostén donde los adolescentes buscan conocimiento para el cuidado de su propia salud corresponde a la familia y amigos, y en menor medida el farmacéutico (Comunalidad 0,515).

**Factor 3:** Asociada a dos variables, el sentirse parte del curso, y sentirse parte del grupo de compañeros, sosteniendo un poder explicativo de la varianza total de 11,35%. La variable el sentirse parte del curso, es explicada por el total de los factores en un 66,5% (Comunalidad 0,665), y representa el 63,68% (0,798²=63,68%) de la varianza total, es decir el 95,75% (0,6368/0,665=95,75%) del total del espacio de los factores. La variable el sentirse parte del grupo de compañeros, queda explicado por el total de los factores en un 65,9% (Comunalidad 0,659), y representa el 62,56% (0,791²=62,56%) de la varianza total, es decir el 94,93% (0,6256/0,659=94,93%) del total del espacio de los factores. En esta dimensión podemos identificar que el sentido de pertenencia con el entorno escolar asume un rol importante para los adolescentes escolarizados.

**Factor 4:** Asociada a dos variables, el posicionar como muy importante la práctica del propio cuidado de la salud y valorar como muy importante a los cuidados de salud en general, sostenido por un poder explicativo de la varianza total de 9,841%. La variable de posicionar importante el propio autocuidado, queda explicado por el total de los factores en un 73% (Comunalidad 0,730), y representa el 70,72% (0,841²=70,72%) de la varianza total, es decir el 96,87% (0,7072/0,730=96,87%) del total del espacio de los factores. La variable de posicionar el cuidado de salud en general, queda explicado por el total de los factores en un 62,2% (Comunalidad 0,622), y representa el 58,82% (0,767²=58,82%) de la varianza total, es decir el 94,56% (0,5882/0,622=94,56%) del total del espacio de los factores. Por lo tanto, en este grupo de adolescentes el autocuidado de la salud se coloca en una posición relevante.

**Factor 5:** Asociada a una variable, el considerar al consumo de alcohol como un serio problema, asume un poder explicativo de la varianza total de 7,143%. La variable de considerar al consumo de alcohol como un serio problema, queda explicado por el total de los factores en un 65,2% (Comunalidad 0,652), y representa el 58,52% (0,765²=58,52%) de la varianza total, es decir el 89,75% (0,5852/0,652=89,75%) del total del espacio de los factores. Variables de consumo de jugos azucarados, explicada por el total de los factores en 59,4% (Comunalidad 0,594), representa el 3,95% (0,629²=3,95%) de la varianza total, delimitando a su vez, a la variable que define el punto de cohorte de la extracción del modelo. En esta dimensión se observa que los adolescentes expresan en la problemática del consumo de alcohol, una preocupación relevante, y en menor cuantía, en el consumo de bebidas azucaradas.

**Tabla 4:** Factores latentes de la muestra identificados mediante Análisis Factorial Explicativo (AFE)

	Matriz de coeficiente de puntuación de componente					
	Variable	Componente				
		F1	F2	F3	F4	F5
1	Importancia del cuidado de salud en general	-0,022	-0,014	-0,108	*0,492*	0,028
2	Poseo prácticas de autocuidado	0,002	-0,002	-0,027	*0,539*	-0,078
3	Habitualmente pienso en positivo	-0,005	-0,149	0,299	0,292	-0,055
4	Me apoyo para cuidar mi salud en el farmacéutico	0,035	0,362	0,096	0,056	-0,129
5	Me apoyo para cuidar mi salud en mi familia	-0,016	*0,44*	-0,013	-0,078	0,101
6	Me apoyo para cuidar mi salud en mis amigos	-0,039	*0,452*	-0,073	-0,049	-0,013
7	Consumo habitualmente jugos en la semana	0,083	-0,131	-0,027	-0,054	0,505
8	Consumo habitualmente cerveza en la semana	*0,355*	-0,053	0,055	0,004	-0,144
9	Consumo habitualmente vino en la semana	*0,369*	0,007	-0,046	0,054	-0,152
10	Consumo habitualmente fernet en la semana	*0,36*	0,044	-0,044	-0,042	-0,025
11	Me siento parte del grupo de compañeros	-0,047	0,073	*0,495*	-0,058	-0,037
12	Me siento parte del curso escolar	0,044	-0,016	*0,504*	-0,11	0,035
13	Consumo habitualmente gaseosas en la semana	0,128	0,018	0,07	-0,091	0,297
14	El consumo de alcohol es un gran problema	-0,198	0,058	-0,033	0,029	*0,696*

Metodo de extraccion: Analisis de componentes principales

Metodo de rotacion: Varimax con normalizacion de Kaiser.

Puntuaciones de componente: Solo se utilizan los casos para los cuales "genero" es femenino en la fase de analisis.

F1: en relacion al consumo habitual

F2: en relacion al apoyo que autorreporta para el cuidado de su salud

F3: en relacion al sentido de pertenencia grupal

F4: en relacion a la importancia que le otorga al cuidado de la salud

F5: en relacion a la mayor preocupacion que tiene el adolescente

Hasta nuestro conocimiento, escasos estudios analizaron asociación entre autocuidado y fecha de último control médico. Si bien se asume en el adolescente una reducida vulnerabilidad natural ante enfermedades, en este estudio el control médico resultó un factor influyente ^23a^ . Igualmente, nuestro hallazgo en relación a los pensamientos positivos, refuerzan la concepción de una relación entre el auto-concepto emocional y satisfacción con la vida, asociándose directamente con el autocuidado ^24^ , lo que incrementa la probabilidad de mantener prácticas de autocuidado. En relación a la toma de decisión asociada al género, autores identificaron que los adolescentes varones sostenían un mayor concepto físico, social y emocional respecto a las niñas ^25^ ; como también describieron una disminución de los conceptos positivos a partir de los 12 años de edad en niñas ^26^ . Nuestro hallazgo sostiene que las niñas adolescentes poseen mayores fortalezas para la toma de decisión que los varones, exhibiendo asimetrías de género en la toma de decisión adolescente, estimado por autores como un factor de riesgo o de protección según conductas

de relación del adolescente con el entorno ^27^ , al igual que el uso de aplicaciones de salud en móvil celular ^28^ . El apoyo de la familia y amigos en el auto-cuidado de salud [F2-F3-F4], como así también el sentirse aceptados por sus compañeros y grupo de amigos, constituyen factores protectores para preservar buena salud adolescente; mientras que un aspecto a profundizar, constituye el consumo de bebidas con riesgos de conducta perjudicial ^23b^ . Los hábitos alimentarios en ambos géneros se asumen como saludables, sin embargo, auto-reportaron consumos frecuentes de bebidas azucaradas y consumos semanales de bebidas con riesgo adictivo, admitiendo preocupación por el consumo de bebidas con contenido alcohólico [F1 – F5]; exhibiendo discordancias entre la auto-percepción del cuidado de salud del adolescente con la práctica del cuidado. Finalmente, si bien estudios sostienen en la medición de actividad física una herramienta útil para comprender en mayor profundidad la autopercepción de salud ^29, 30^ , en esta población no resultó indicativo. Se destaca la predisposición para modificar prácticas por otras más saludables.

## Conclusión

El auto-cuidado de salud en adolescentes de esta región, es asimétrico en cuanto al género, y se identifican discordancias entre la auto-percepción con la práctica. El uso de aplicaciones digitales en salud, el efectuar controles médicos con frecuencias anuales o menor, y el mantener hábitos de pensamientos positivos, incrementan la probabilidad de sostener prácticas regulares del autocuidado de salud. Las niñas poseen mayor solvencia en la toma de decisión para resolver conflictos con sus compañeros, de pensar antes de actuar, de eliminar con facilidad sentimientos de rencor; y en cuidar más de su salud. Existe preocupación entre los adolescentes del consumo de bebidas con contenido etílico.

## References

[1] Organización Panamericana de la Salud. Un nuevo análisis de las Naciones Unidad revela un deterioro alarmante de la salud en las mujeres, los niños y los adolescentes. 2022. Disponible en: https://www.paho.org/es/noticias/18-10-2022-nuevo-analisis-naciones-unidas-revela-deterioro-alarmante-salud-mujeres-ninos

[2] Organización Mundial de la Salud. Comunicados de prensa. Berlín. 2022. Disponible en: https://www.who.int/es/news/item/18-10-2022-staggering-backsliding-across-women-s--children-s-and-adolescents--health-revealed-in-new-un-analysis

[3] Organización Panamericana de la Salud. La salud en las Américas. Estado de salud de la población. La salud de los adolescentes. 2017. Disponible en: https://www.paho.org/salud-en-las-americas-2017/ro-adolescent-es.html

[4] Ministerio de Salud de la Nación. Argentina. Sistema Estadístico de Salud. Indicadores de Niñez y Adolescencia. Aspectos demográficos Argentina 2021. Disponible en: https://www.argentina.gob.ar/sites/default/files/2021/12/senaf_dngdi-indicadores_nna-aspectos_demograficos-arg2021_25_abr_2022.pdf

[5] Análisis de Situación de la Niñez y la Adolescencia en Argentina. Resumen ejecutivo. UNICEF Argentina. Buenos Aires. Primera Ed. junio 2021. Disponible: https://www.unicef.org/argentina/media/12121/file/SITAN%20-20Resumen%20Ejecutivo.pdf

[6] Ministerio de Salud. Argentina. Segundo Informe de Indicadores Priorizados – Julio 2022. ENNyS2. Disponible en: https://bancos.salud.gob.ar/recurso/segundo-informe-de-indicadores-priorizados-julio-2022-ennys-2

[7] Indicadores de Niñez y Adolescencia. Secretaria Nacional de Niñez, Adolescencia y Familia. Ministerio de Desarrollo Social. Argentina. p.61. 2021. Disponible en: https://www.argentina.gob.ar/sites/default/files/2021/12/senaf_dngdi-indicadores_nna-mortali dad_de_0_a_19_anos-arg2019_25_abr_2022.pdf

[8] Maceira D, Ryan D, Fuster V. Hacia un sistema alimentario más saludable y sostenible en Argentina: informe de análisis de actores. Repositorio digital. CEDES. 02-2022. [citado 14 de diciembre de 2022] Disponible en: https://idl-bnc-idrc.dspacedirect.org/handle/10625/61690

[9] Navarro Peña Y, Castro Salas M. Modelo de Dorothea Orem aplicado a un grupo comunitario a través del proceso de enfermería. Enf. globo. [citado 06 de febrero de 2023] Disponible en: http://scielo.isciii.es/scielo.php?script=sci_arttext&pid=S1695-61412010000200004&lng=es

[10] Orem DE, Taylor SG, Renpenning KML. BOOK Nursing: Concepts of Practice. SN - 9780323008648. UR – Mosby. 2001. Disponible en: https://books.google.com.ar/books?id=YR1tAAAAMAAJ

[11] Alfaro RL, Esquivel RN, Paredes ER, Muñoz CL. Autopercepción de la imagen corporal y hábitos alimenticios en estudiantes universitarios, Arequipa 2012. Revista de la Facultad de Medicina Humana. 2015; 15(3), 8-13.

[12] La Banca RO, Nascimento LC. Posicionar a los niños en el centro de su cuidado: reflexiones sobre el desarrollo cognitivo y la alfabetización en salud infantil. Revista da Escola de Enfermagem da USP. 2019; 53. 10.1590/S1980-220X2019ed0303533 31800824

[13] Morales Aguilar R, Flórez Flórez ML. Agencia de autocuidado y factores de riesgo cardiovascular en adolescentes. Avances en Enfermería. 2016; 34(2), 125-136.

[14] Lee EW, Kim HS, Yoo BN, Lee EJ, Hyun Park J. Effect of a Primary Care-Based Chronic Disease Management Program for Hypertension Patients in South Korea. Iran J Public Health. 2022 Mar;51(3):624-633. doi: 10.18502/ijph.v51i3.8939. 10.18502/ijph.v51i3.8939 PMC9276591 35865058

[15] Boraita R. Valoración del estado de salud física y psicosocial en los adolescentes de La Rioja. Tesis de doctorado, Universidad de La Rioja. España. 2021.

[16] Cabezas Henríquez MF. Alfabetización alimentaria, autorregulación alimentaria y su asociación con la dieta, estado nutricional y bienestar de adultos en Chile. 2021. Disponible en: http://repositorio.udec.cl/handle/11594/6692

[17] Vilchez NV, Sandoval AF, Chacana HG, Fuentes CC, Yañez SM, Gálvez CP, Aro CP. Efecto de la danza en la mejora de la autoestima y el auto-concepto en niños, niñas y adolescentes: Una revisión. Retos: nuevas tendencias en educación física, deporte y recreación. 2021; (40), 385-392.

[18] Evelyn GB, Yasser GB. La prevención de adicciones en la adolescencia desde un enfoque de autocuidado. [disertación]. In V Simposio académico sobre adicciones. Cedro 2022. Universidad de Matanzas, Cuba.

[19] Rojas-Barahona CA, Zegers B. y Förster CE. La escala de autoestima de Rosenberg: Validación para Chile en una muestra de jóvenes adultos, adultos y adultos mayores. Revista médica de Chile. 2009; 137 (6), 791-800. 19746281

[20] Palenzuela-Luis N, Duarte-Clíments G, Gómez-Salgado J, Rodríguez-Gómez JÁ, Sánchez-Gómez MB. Cuestionarios que evalúan el auto-concepto, la autopercepción, la actividad física y el estilo de vida de los adolescentes: una revisión sistemática Niños. 2022; 9 (1), 91.

[21] Navarro Peña Y, Castro Salas M. Modelo de Dorothea Orem aplicado a un grupo comunitario a través del proceso de enfermería. Enfermería global. 2010; 9(2):1-14. Disponible en: https://www.redalyc.org/comocitar.oa?id=365834755004

[22] Martín AE. Relativismo de género: cómo el contexto da forma a lo que se ve como masculino y femenino. Revista de Psicología Experimental: General. 2023; 152 (2), 322.

[23] Carrillo-Ramírez E, Pérez-Verduzco G, Laca-Arocena FA, Luna-Bernal AC. Inteligencia emocional percibida y auto-concepto en adolescentes estudiantes de bachillerato. Revista de Educación y Desarrollo. 2020; 55, 33-40.

[24] Palacios EG, Echaniz IE, Rodríguez Fernández A, Ortiz de Barrón IC. Autoconcepto personal y satisfacción con la vida en la adolescencia, juventud y adultez". Psicothema 27.1 2015; 52-58. Disponible en: http://psiqu.com/2-49803 10.7334/psicothema2014.105 25633770

[25] Plangger L, Rodríguez Quintana E, González CU. Diferencias interculturales en el autoconcepto: dimensión estabilidad emocional, en adolescentes. Universidad Complutense de Madrid. Revista de pedagogía. 2018. Disponible en: https://recyt.fecyt.es/index.php/BORDON/article/view/57854/39320

[26] Cardona MNT. Auto concepto y Estilos de Aprendizaje en un grupo de niños colombianos de tercero a quinto Grado de escuela primaria. Tesis Doctoral. Universidad de Montemorelos. México. Repositorio institucional. 2016. Disponible en: https://dspace.um.edu.mx/handle/20.500.11972/712

[27] Bello Pulido JC, Hurtado Nieto PR, Villalba Yibirin ZE, Moreno Méndez JH. El papel de las competencias emocionales parentales en las conductas internalizantes y el auto concepto de los niños. Psicogente. 2020: 23(44), 166-188.

[28] Pérez-Sánchez R, Dodel M. Predictores del uso problemático del teléfono celular en adolescentes costarricenses. Revista Latinoamericana de Ciencias Sociales, Niñez y Juventud. 2023; 21(1), 1-21.

[29] Gómez Baya DM, Mendoza AR. La actividad física como factor de protección para el desarrollo a lo largo del ciclo vital. La promoción de la actividad física en la sociedad contemporánea: orientaciones para la práctica profesional. Ed. Díaz de Santos. 2003.

[30] Cuesta AIO, Mínguez LAM, Peláez MS, Solana JF. La influencia del profesor de Educación Física en la automotivación y la autoestima a través de la práctica de ejercicio físico durante la adolescencia. In Cuestiones relativa a la inclusión de colectivos vulnerables. Dykinson. 2022; 101-13.

